# Deep learning for classification of aggressive versus non-aggressive central giant cell granuloma using whole-slide histopathology images

**DOI:** 10.1007/s00428-025-04160-z

**Published:** 2025-06-27

**Authors:** Marilena Vered, Anna Shnaiderman-Shapiro, Rozet Malouf, Ariel Hirschhorn, Amos Buchner, Shoshana Reiter, Lazar Kats

**Affiliations:** 1 Department of Oral Pathology, Oral Medicine and Maxillofacial Imaging, School of Dentistry, Tel Aviv University, 69978 Tel Aviv, Israel; 2 Institute of Pathology, Sheba Medical Center, Tel Hashomer, Israel; 3 Department of Cranio‐Maxillofacial Surgery, Chaim Sheba Medical Center, Tel Hashomer, Israel

**Keywords:** Central giant cell granuloma, Aggressive, Non-aggressive, Deep machine learning model

## Abstract

Microscopic images of aggressive and non-aggressive cases of central giant cell granuloma (CGCG) were analyzed by deep learning algorithms in order to assess its potential as a tool in predicting the biological behavior of CGCG. CGCGs with cortical expansion/perforation, tooth resorption/displacement, or recurrence were classified as aggressive (A-group; *N* = 48), CGCGs without these features as non-aggressive (N-group; *N* = 39). Data on patient age, gender, and jaw location were collected. Hematoxylin–eosin (H&E)–stained sections were scanned at × 10 magnification, yielding 9982 sections (5236 A-group, 4746 N-group). After excluding artifacts, 4272 sections (2629 A-group, 1643 N-group) were used to train a ResNet-50 model pre-trained on ImageNet. Data augmentation included random rotation, flipping, and zooming. Model was trained for 100 epochs with an 80/20 train/validation split and tested on 100 images (50 A-group, 50 N-group). Receiver Operating Characteristic (ROC) analysis with area under the curve (AUC), sensitivity, and specificity was performed; *t*-test and chi-square test were used for age and frequency (*p* < 0.05). AUC was 52%, sensitivity 54%, and specificity 50%. Mean age of patients in A-group was lower than in N-groups (32.6 ± 19.98 years and 42.2 ± 21.58 years, respectively; *p* = 0.038). F:M ratio was 1:1 in both groups. Mandible was twofold more frequently than maxilla in both groups. This pioneering study to differentiate between aggressive and non-aggressive CGCGs based on whole microscopic sections using a deep machine learning model was not successful, probably due to lack of specific segmentations and technical staining issues. Further investigation with advanced preprocessing is needed to enhance model performance and clinical utility.

## Introduction

Central giant cell granuloma (CGCG) is defined as a localized, benign but sometimes aggressive osteolytic jaw lesion consisting of a proliferation of osteoclasts in a mononuclear and fibrous vascular stroma [1]. The incidence of CGCG is 1.1 per million in Europe [2] and constitutes about 10% of the benign jawbone lesions [3].

Most cases occur in females and in patients aged < 30 years, with a propensity to the anterior mandible [4]. Clinically, most CGCGs are considered *non-aggressive* lesions as they are typically asymptomatic, slow-growing, painless swellings without perforation or root resorption and usually do not recur after conservative removal. On the other hand, about one-third of CGCGs are *aggressive* lesions as they are painful, fast-growing, and lead to erosion of the bones, cortical expansion and perforation, resorption of the adjacent roots, tooth displacement, and may spread into the soft tissues and have a tendency to recur after curettage [3, 5, 6]. Accordingly, non-aggressive CGCG is treated by conservative surgery, whereas aggressive CGCG usually necessitates a more radical approach [1].

Microscopically, CGCG either aggressive or non-aggressive is an unencapsulated proliferation of mononuclear spindle-shaped and polygonal cells intermingled with osteoclast multinucleate giant cells in a vascular background, with hemorrhage and hemosiderin pigment. The giant cells in CGCGs show reactivity to osteoclast and macrophage markers [3, 7, 8]. The mononuclear stromal cells are the proliferating component and mitoses may be readily found.

Predicting the biological behavior of CGCGs, aggressive/non-aggressive, has been the focus of many clinical, histomorphologic, and molecular studies along the years. To mention just a few, molecular markers for macrophages (e.g., CD68, CD163, CD115), vascularity/angiogenesis (e.g., CD31, CD34, WT-1, CD105, D2-40), stromal myofibroblasts (alpha-smooth muscle actin), and proliferation markers (e.g., Ki67) did not reveal any concrete discriminating prognostic marker [9–12]. Young age seems to be associated with aggressive CGCGs [12].

CGCGs were recently found to be driven by mutually exclusive somatic mutations in *KRAS*, *FGFR1*, and *TRPV4* genes that occur in 70% of cases leading to MAPK-pathway activation [13, 14]. Unlike giant cell tumors of long bones, *H3F3A* mutations are not present [14, 15].

Machine learning techniques are present in many areas of modern society. These applications increasingly leverage a class of techniques called deep learning, which are also widely used in various fields of medicine [16]. Models based on these methods have also been used in dental research, including diagnosis of caries and periodontal and endodontic lesions [17]. Research on the possible application of deep learning algorithms in histopathological diagnosis has received great attention in the medical community, as well [18, 19]; these algorithms were also used in an attempt to aid in diagnosis of precancerous and cancerous lesions in the head and neck [20]. To our knowledge, no peer-reviewed studies have been published that investigate how this approach can be used to differentiate between aggressive and non-aggressive CGCG cases.

This study aims to develop a deep learning model (ResNet-50) to classify aggressive and non-aggressive CGCGs based on H&E-stained microscopic images, hypothesizing that the model can achieve satisfactory sensitivity and specificity for predicting biological behavior. By automating the differentiation of CGCG subtypes, this research seeks to enhance diagnostic precision, reduce the risk of overtreatment or undertreatment, and pave the way for future AI-driven tools in oral pathology, ultimately improving patient outcomes in a field where diagnostic uncertainty remains a significant barrier.

## Material and methods

### Study design

This retrospective study was performed with the approval of the Tel Aviv Ethics Committee #5360–2. In writing this study, the main relevant principles of Checklist for Artificial Intelligence in Medical Imaging have been followed [21].

The biopsy files of the laboratory of the Dept. of Oral Pathology were searched for cases of CGCG, 2000–2022 (part of the present cases were part of a previous study on CGCG [12]. Cases with known previous treatment with calcitonin, corticosteroids, or any other agent, and cases with a diagnosis of hyperparathyroidism or other giant cell–containing type of lesion, were excluded from the study.

### Data sources

Cases were sourced from the Dept. of Oral Pathology laboratory archives. Data on patient age, gender, lesion location, size, cortical expansion/perforation, tooth resorption/displacement, and recurrence were collected anonymously. Lesions were classified as aggressive (A-group: cortical expansion/perforation, tooth resorption/displacement, or recurrence) or non-aggressive (N-group: none of these features).

### Data preprocessing

**Fig. 1:**
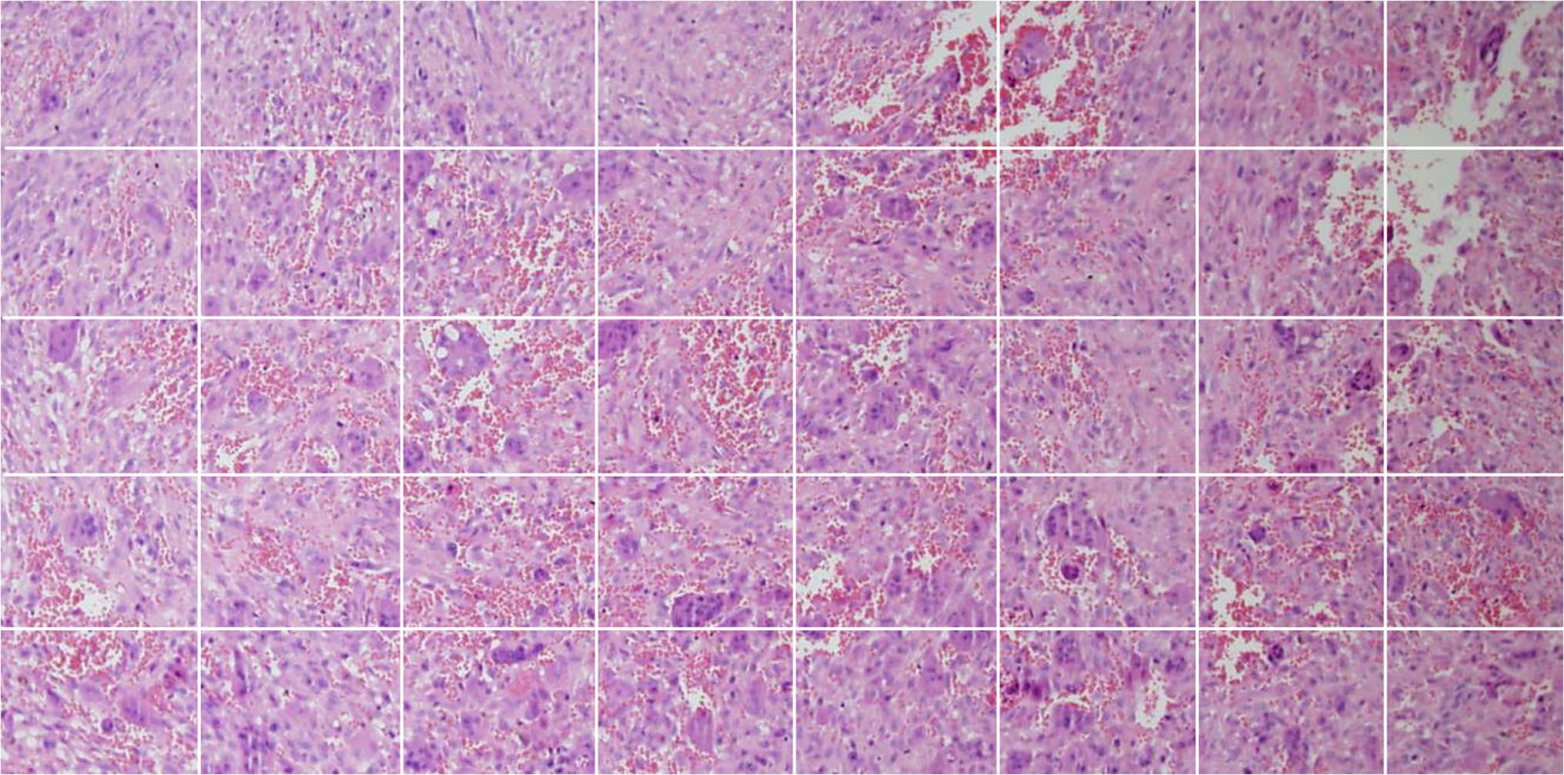
Example of scanned image of a CGCG case

H&E-stained slides were scanned at × 10 magnification using a motorized microscope (BS2085, BestScope, Beijing, China). Images were stitched into whole-slide composites using proprietary software (BestScope, v2.1.3) (Fig. 1). Regions of interest (ROIs) containing CGCG tissue were manually segmented. Preprocessing included normalization to 224 × 224 pixels (ResNet-50 input size) and data augmentation (random rotation ± 30°, horizontal/vertical flipping, zooming 0.8–1.2 ×). We opted not to apply stain normalization methods to the original H&E-stained slides to preserve the inherent variability in staining patterns, which may reflect subtle histopathological differences between aggressive and non-aggressive CGCGs. Stain normalization, while reducing color variability, risks masking biologically relevant features, such as variations in cellular uptake or tissue composition, that could be critical for distinguishing CGCG subtypes. Given the histological similarity between aggressive and non-aggressive CGCGs, retaining the original slide characteristics was hypothesized to enhance the ResNet-50 model’s ability to detect nuanced patterns, despite the potential for staining variability to introduce noise. This decision aligns with exploratory studies in histopathology where raw image data are prioritized to capture all possible diagnostic cues. Sections with artifacts (e.g., non-CGCG tissue, empty fields, or uneven staining obscuring tissue) were excluded using visual inspection by two oral pathologists, reducing the dataset from 9982 to 4272  sections (2629 A-group, 1643 N-group). Preprocessing was performed using OpenCV (v4.5.3, default settings) and NumPy (v1.19.2, default settings).

### Data partitions

Data were split into training (80%, 3418 sections), internal testing (20%, 854 sections), and test (100 images, 50 A-group, 50 N-group) sets. Partitions were disjoint at the patient level to prevent data leakage. The 80/20 split was chosen to maximize training data while ensuring sufficient internal testing, following common practice in deep learning studies [17]. The imbalance between A-group (2629 sections) and N-group (1643 sections) was addressed by oversampling the N-group during training to balance class representation.

### Exclusion criteria

**Fig. 2:**
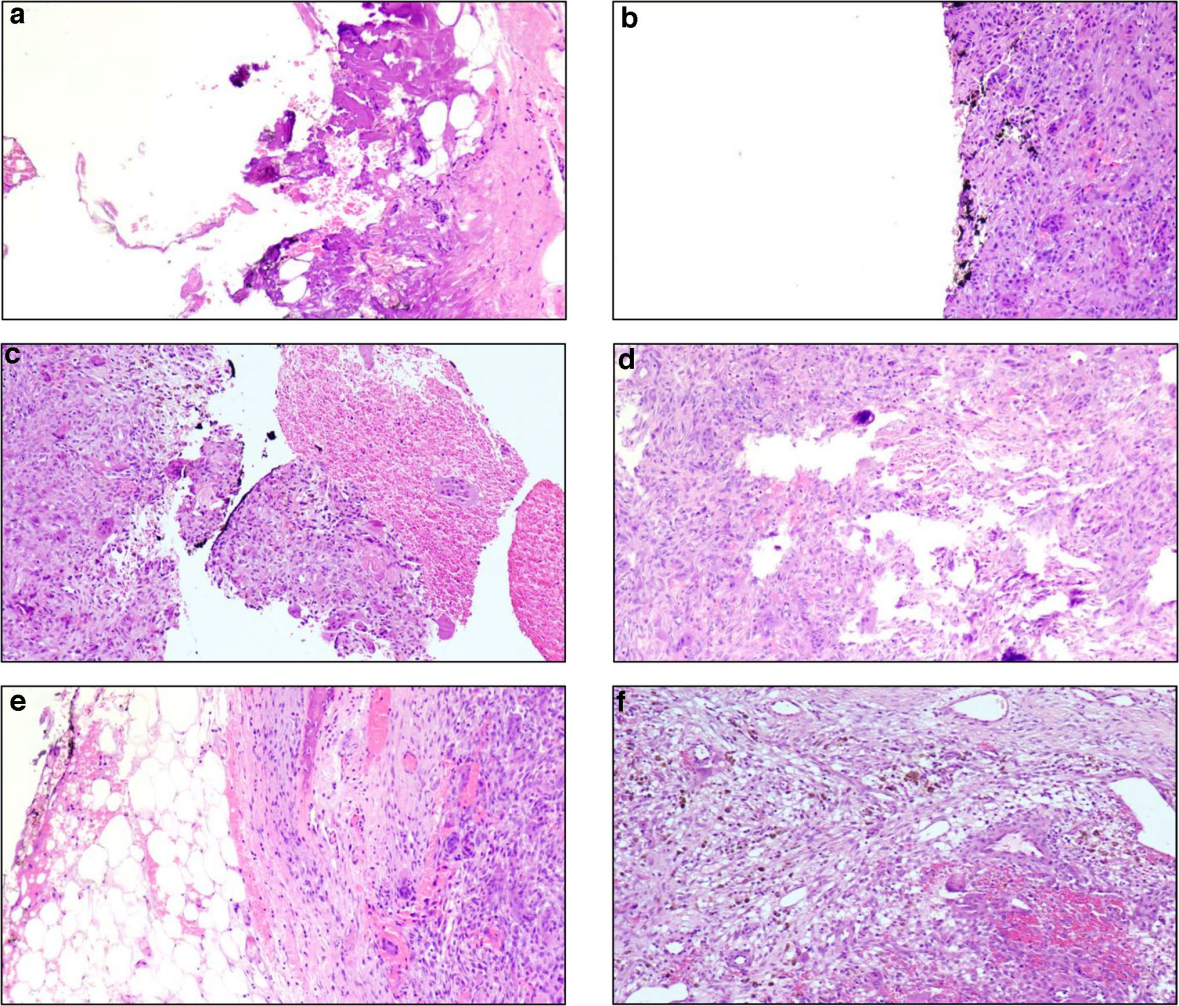
Examples of sections that were excluded from study. **a** Marginal field with thermal artifacts; **b** only the right area shows CGCG, and the rest is empty field; **c** fragmented biopsy of CGCG with empty spaces and hemorrhagic areas; **d** CGCG with sectioning artifacts; **e** only the right part of the field is CGCG, and the rest is connective tissue and adipose tissue; **f** only the right lower corner of the field is CGCG, and the rest is connective tissue with inflammation

Sections showing artifacts such as those caused by excessive or uneven distribution of staining, tumor-free areas at the edges of the scanned fields or within sections, and areas containing tissues other than CGCG were all excluded from the study (Fig. 2).

### De-identification

Patient data were anonymized by removing identifiable information (e.g., names, medical record numbers) before analysis, per GDPR guidelines.

### Missing data

Cases with incomplete clinical data (e.g., missing recurrence status) were excluded during case selection. No imputation was performed for image data.

### Reference standard

The reference standard was clinical classification (aggressive vs. non-aggressive) based on cortical expansion/perforation, tooth resorption/displacement, or recurrence, as documented in patient records. This standard was chosen due to its clinical relevance and the absence of reliable molecular markers for CGCG [5]. Limitations include potential subjectivity in recurrence reporting and variability in clinical documentation, which may introduce bias, particularly if aggressive features were underreported in older records [4]. Clinical classifications were assigned by two oral pathologists reviewing patient records independently. Discrepancies were resolved by consensus. No additional image annotations were performed, as whole-slide images were used.

### Annotation tools

Clinical data were extracted using Microsoft Excel (v16.0). ImageJ was used for ROI segmentation.

### Inter-rater variability

Cohen’s kappa was calculated for inter-rater agreement on clinical classification (kappa = 0.85, indicating strong agreement). Discrepancies were resolved via discussion.

### Neural networks architecture

A deep neural network, ResNet-50, was used, pre-trained on the ImageNet dataset using transfer learning. The ResNet-50 model, a 50-layer convolutional neural network, was used with pre-trained ImageNet weights. Inputs were 224 × 224 × 3 RGB images; outputs were binary probabilities (A-group vs. N-group). The architecture included convolutional layers, batch normalization, ReLU activations, and global average pooling, followed by a fully connected layer with softmax activation. Modifications included a custom classification head.

### Software

The model was implemented using Keras (v2.4.3) with TensorFlow (v2.3.0) backend, running on an NVIDIA GeForce GTX 1080 Ti GPU. Image preprocessing used OpenCV (v4.5.3) and NumPy (v1.19.2).

### Software

*Initialization*: Weights were initialized via transfer learning from ImageNet, except for the classification head, which was randomly initialized using Glorot uniform distribution.

### Training

Training used stochastic gradient descent (learning rate = 0.001, momentum = 0.9), batch size = 32, and categorical cross-entropy loss. Data augmentation was applied as described. Training ran for 100 epochs, with early stopping if validation loss plateaued for 10 epochs. The best model was selected based on validation AUC.

### Model selection

The model with the highest validation AUC was chosen for testing.

### Ensembling

No ensembling was used.

### Evaluation metrics

Performance was assessed using AUC, sensitivity, specificity, and ROC analysis. Confidence intervals (95%) were calculated using DeLong’s method. Statistical significance was tested with z-statistics (*p* < 0.05).

### Robustness analysis

Sensitivity to staining variability was tested by retraining on subsets with standardized vs. non-standardized images, showing no significant AUC improvement (*p* = 0.72).

### Explainability

Gradient-weighted Class Activation Mapping (Grad-CAM) was used to visualize regions influencing model predictions, highlighting giant cell clusters. Validation was qualitative, comparing Grad-CAM outputs to pathologist annotations.

### Test data

The test set (100 images) was drawn from the same dataset but disjoint at the patient level. No structural differences existed between training and test sets.

### Statistical analysis

Receiver Operating Characteristic (ROC) analysis was performed for the machine learning analysis. Null hypothesis: true area = 0.5, confidence interval (CI) 95%. The MedCalc Statistical Software version 14.8.1 (MedCalc Software Ltd, Ostend, Belgium) was used for the statistical analysis. Metrics for statistical analysis: ROC analysis, Sensitivity, Specificity.

Additional tests included *t*-test and chi-square for continuous parameters (age) and categorical parameters (frequency). Significance was set at *p* < 0.05.

## Results

### Demographics

A-group comprised 48 patients with 84 scanned slides. Age range of the patients was 3–82 years, mean 32.6 ± 19.98 years.

There were 24 males and 24 females with a 1:1 gender ratio.

Sixteen tumors were located in the maxilla and 32 in the mandible, with a maxilla:mandible ratio of 1:2.

N-group comprised 39 patients with 47 scanned slides. Age range of the patients was 5–76 years, mean 42.2 ± 21.58 years. N-group patients were significantly older than A-group patients, *p* = 0.038.

There were 19 males and 20 females with a 1:1 gender ratio.

Thirteen tumors were located in the maxilla and 26 in the mandible, with a maxilla:mandible ratio of 1:2.

**Fig. 3:**
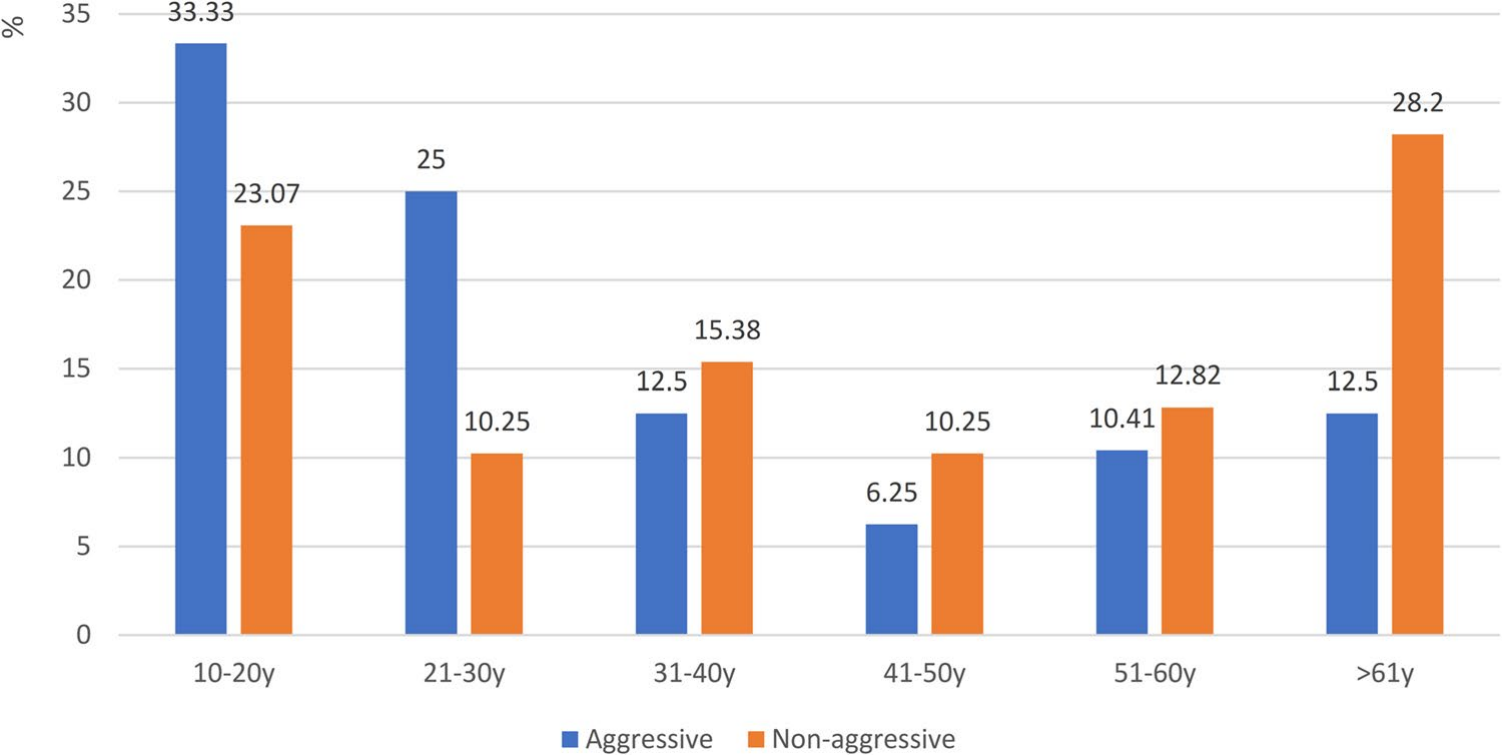
Distribution of patients (%) among decades of life

The distribution of patients (%) among decades of life is shown in Fig. 3. A-group patients are more frequent than N-group patients during the first three decades of life, while the N-gorup patients are more frequent than A-group patients from the 4th decade of life and on. Potential biases include limited demographic diversity (e.g., lack of race/ethnicity data due to retrospective record limitations) and age skew, which may affect generalizability.

### Machine learning outcomes

**Fig. 4:**
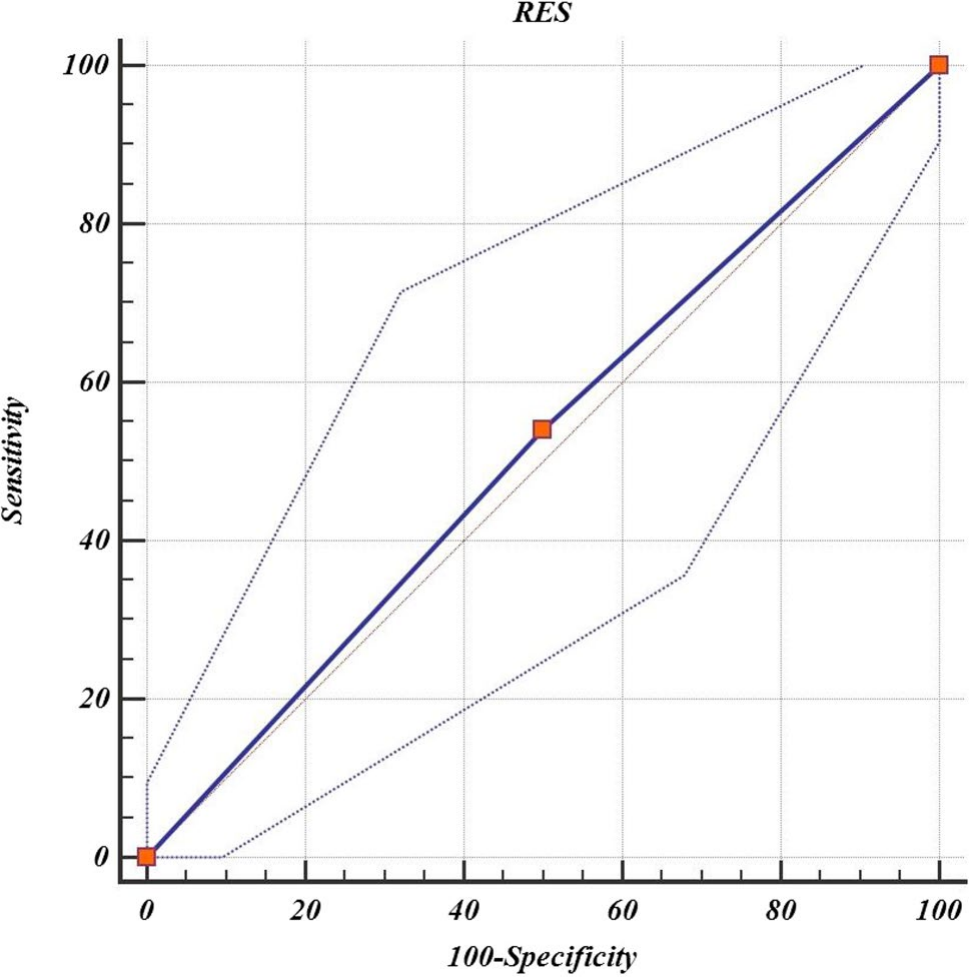
ROC curve, AUC 52%

Model performance: On the test set, AUC = 0.52 (95% CI = 0.421–0.619, *p* = 0.69), sensitivity = 54%, specificity = 50%, and accuracy = 52% (Fig. 4). Performance was equivalent to random guessing. No significant differences were found compared to baseline (*p* > 0.05).

## Discussion

The aim of this study, the first of its kind, to the best of our knowledge, was to use algorithms based on machine learning methods to analyze and classify the microscopic images of aggressive and non-aggressive cases of CGCG in order to assess its potential as a tool in predicting the biological behavior of CGCGs. We hypothesized that deep learning–based algorithm has the potential to distinguish between aggressive and non-aggressive CGCGs with a satisfactory sensitivity and specificity based on the general classification of the lesion area. Results showed that the algorithm did not reach sensitivity or specificity values required for differentiation between aggressive and non-aggressive lesions of CGCG, refuting study hypothesis. Only the age of the patients seemed to be a factor that can be related to the biological behavior of the lesions, with young patients (1st–3rd decades of life) being more likely to have aggressive biological behavior in comparison to older patients (4th decade and on).

We have found that all CGCG lesions in both the A-group and N-group were more frequent (twofold) in the mandible than in the maxilla and this is in conform with other studies [4, 9] and with one of our previous studies [12] but not with another of our studies [11]. We did not find a higher prevalence of CGCG in females than in males in the present study or in previous studies from our department based on various sizes of the study groups [11, 12], in contrast to other studies, in which a female prevalence was reported [4, 6, 9]. Collectively, it is shown that demographic data on jaw location of CGCGs and gender propensity might be study-related and should not be considered unequivocal features. In contrast, age distribution of aggressive CGCGs seems to be a more consistent finding but, yet, not absolute. We presently found that the aggressive lesions were more common in patients in their first 3 decades of life, which is in accordance with other studies [5, 6, 11, 12, 22], but in contrast to the study by Melo-Muniz et al. [9].

In line with the lack of any substantial and consistent morphological and molecular ability to differentiate between aggressive and non-aggressive CGCGs, as reported in studies based on pathologists’ visual interpretation, the algorithm that was presently used also did not have the ability to distinguish between the aggressive and non-aggressive CGCGs. To the best of our knowledge, there are no similar studies on CGCG at a microscopic level. Giant cell tumor (GCT) of bone is essentially microscopically similar to CGCG, although it has been shown to be genetically different [10]. There are several studies in which machine learning algorithms were employed to identify this tumor and differentiate it from adjacent normal bone tissues or other bone tumors based on radiologic features (radiomics) [23–25]. We found only one study where a deep learning model, based on EfficientNet-B3 algorithm, was used to differentiate between classes of primary bone tumors (e.g., chondrogenic tumors, osteogenic tumors, osteoclastic giant cell-rich tumors, other mesenchymal tumors of bone) at a microscopic level [26]. In that study, it was found that the model provided high average AUCs indicating effective classification of histological types of the examined tumors and the authors concluded that this could assist radiologists in achieving better classification results than their mere visual assessment.

**Fig. 5:**
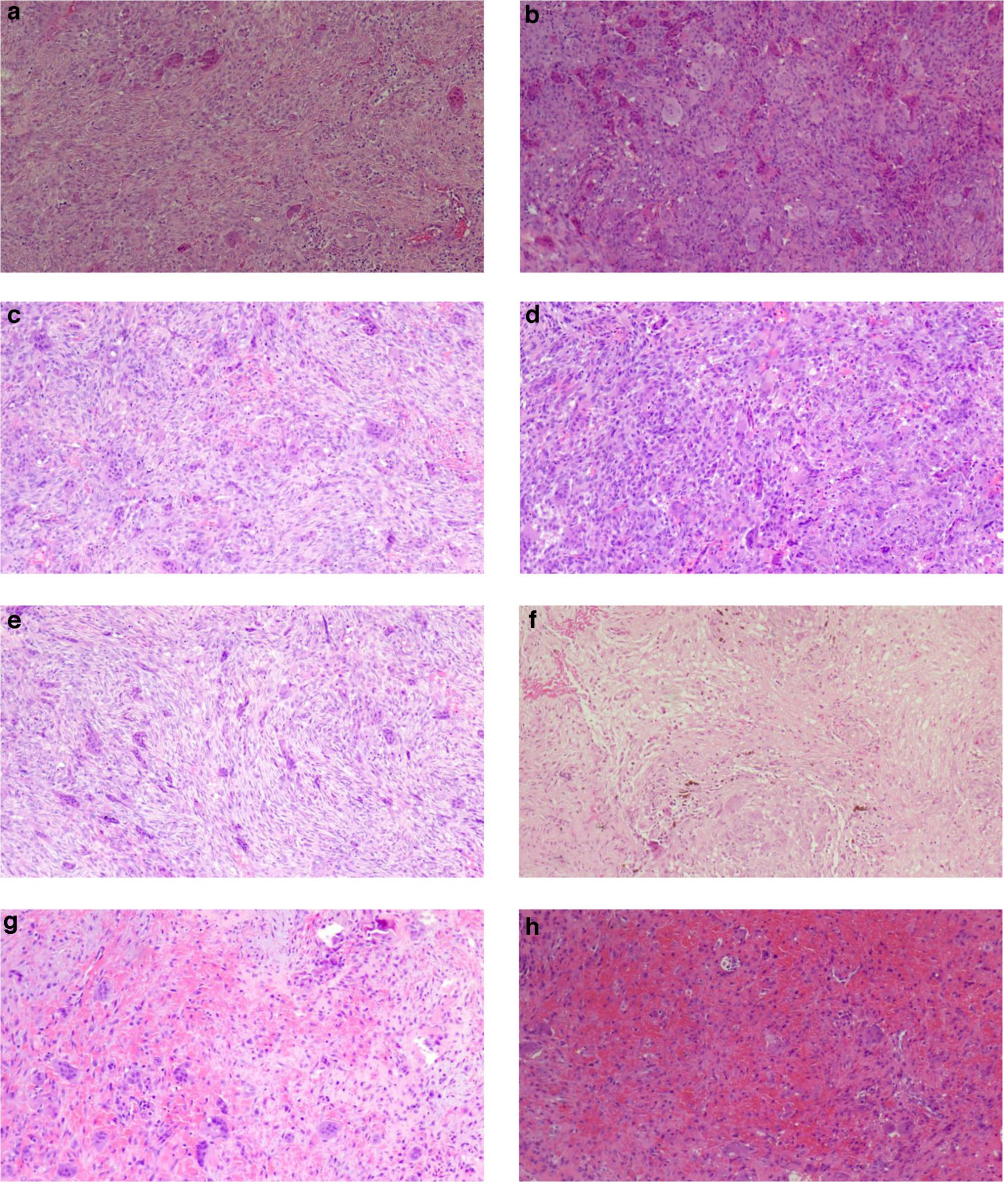
Sections from different CGCGs that highlight the variability in the hues of the hematoxylin and eosin (H&E) stains as a factor of varying distribution of the cellular/non-cellular components among tumors and of the technique-related variations. **a** and **b** These tumors have a relatively similar distribution of mono- and multi-nucleated cells (predominated by the latter), but H&E stain hues differ. **c** and **d** H&E stain hues are similar; however, tumor in **c** is predominated by mononuclear cells while the tumor in **d** by multi-nucleated cells. **e** and **f** These tumors have a relatively similar distribution of scattered mono- and multi-nucleated cells and occasional hemorrhagic areas; H&E stain hues are considerably different. **g** and **h** These tumors have a relatively similar distribution of scattered mono- and multi-nucleated cells with considerable hemorrhagic areas; H&E stain hues are considerably different

At this time point, we can raise two main reasons to explain the lack of success of the algorithm to provide better outcomes. First, the figures submitted to the algorithmic analysis contained the entire range of histomorphologic components including mononuclear spindle cells, multi-nucleated giant cells, various inflammatory cells, endothelial cells, and extravasated red blood cells. It can be assumed that preparing the figures by segmentation of individual cells (at least the mono- and multi-nuclear cells) may have a positive impact on the ability of the algorithm to differentiate between the aggressive and non-aggressive types of CGCG. The second reason that has to be discussed is related to the considerable variability in the spatial relations between the main components of CGCGs, i.e., mononuclear cells, multi-nuclear cells, and hemorrhagic areas with/without hemosiderin deposits (Fig. 5). The hematoxylin and eosin hues of tumors can absolutely differ as a factor of the tumor being predominated by giant cells or by mononuclear cells or by hemorrhagic areas and this might affect the algorithm when the entire figure is submitted for analysis. An additional important factor to consider is at the technical level and it refers to the variability in the hues of the hematoxylin and eosin stains, even if done by the same technician and tissues are being prepared in the same protocol by the same technicians or automated apparatus, nonetheless if sections are prepared in various laboratories. Our decision to forgo stain normalization was intended to preserve potential diagnostic features and avoid any amplified impact of staining variability on the model performance.

In conclusion, our pioneering trial to differentiate between aggressive and non-aggressive CGCGs based on whole microscopic sections using a deep machine learning model was not successful, probably due to section preparation (no segmentations) and technical issues (H&E staining variations). More investigation is needed in order to prepare the sections for analysis in a more unique manner so that the algorithm will be able to produce better outcomes.

## Data Availability

The datasets generated during and/or analyzed during the current study are available from the corresponding author on reasonable request.

## Code availability

English language editing has been performed by the use of GROK, an AI assistant created by xAI.
